# Prognostic factors for elderly gastric cancer patients who underwent gastrectomy

**DOI:** 10.1186/s12957-021-02475-0

**Published:** 2022-01-07

**Authors:** Shunji Endo, Tomoki Yamatsuji, Yoshinori Fujiwara, Masaharu Higashida, Hisako Kubota, Hideo Matsumoto, Hironori Tanaka, Toshimasa Okada, Kazuhiko Yoshimatsu, Ken Sugimoto, Tomio Ueno

**Affiliations:** 1 Department of Digestive Surgery, Kawasaki Medical School, 577 Matsushima, Kurashiki, Okayama, 701-0192 Japan; 2 Department of General Surgery, Kawasaki Medical School, Okayama, Japan; 3 Department of Surgery, Mitsugi General Hospital, Hiroshima, Japan; 4 Department of General Geriatric Medicine, Kawasaki Medical School, Okayama, Japan

**Keywords:** Gastric cancer, Aged, Octogenarians, Surgery, Prognostic factor

## Abstract

**Background:**

Patients with gastric cancer are aging in Japan. It is not clear which patients and which surgical procedures have survival benefits after gastrectomy. A multivariate analysis was performed.

**Methods:**

The medical records of 166 patients aged ≥ 80 years who underwent gastrectomy without macroscopic residual tumors were retrospectively reviewed. Univariate and multivariate analyses using Cox proportional hazard models were performed to detect prognostic factors for overall survival.

**Results:**

In univariate analyses, age (≥ 90 vs. ≥ 80, < 85), performance status (3 vs. 0), American Society of Anesthesiologists physical status (ASA-PS) (3, 4 vs. 1, 2), Onodera’s prognostic nutritional index (< 40 vs. ≥ 45), the physiological score of the Physiological and Operative Severity Score for the enUmeration of Mortality and morbidity (POSSUM) (≥ 40 vs. ≥ 20, ≤ 29), surgical approach (laparoscopic vs. open), extent of gastrectomy (total, proximal vs. distal), extent of lymphadenectomy (D1 vs. ≥ D2), pathological stage (II–IV vs. I), and residual tumor (R1 vs. R0) were significantly correlated with worse overall survival. Multivariate analysis revealed that ASA-PS [3, 4 vs. 1, 2, hazard ratio (HR) 2.30, 95% confidence interval (CI) 1.24–4.24], extent of gastrectomy (total vs. distal, HR 2.17, 95% CI 1.10–4.31) (proximal vs. distal, HR 4.05, 95% CI 1.45–11.3), extent of lymphadenectomy (D0 vs. ≥ D2, HR 12.4, 95% CI 1.58–97.7), and pathological stage were independent risk factors for mortality.

**Conclusions:**

ASA-PS was a useful predictor for postoperative mortality. Gastrectomy including cardia is best avoided.

## Background

The population of Japan is aging year by year. According to official Japanese statistics, 9.3% of Japan's population was estimated to be ≥ 80 years in 2021 [1]. With the aging trend of the population, patients with gastric cancer are also aging. According to the National Clinical Database (NCD) in Japan, the percentages of patients aged ≥ 80 years who underwent distal and total gastrectomy increased from 18.5% and 15.0% in 2011 to 24.0% and 20.9% in 2018, respectively [2].

Some reports showed that the postoperative survivals were equivalent between those aged ≥ 80 years and those aged < 80 years [3–5], while they were worse in the former patients than in the latter patients [6, 7] as elderly patients have reduced physical, physiological, nutritional, and mental functions, comorbidities, and short life expectancy. Furthermore, gastrectomy may reduce oral intake and thus induce malnutrition. Treatment strategies should therefore be carefully selected in consideration of their condition, symptoms, cancer stage, and social support.

Generally, Eastern Cooperative Oncology Group performance status (ECOG-PS) [8] and American Society of Anesthesiologists physical status (ASA-PS) [9] are used for preoperative risk assessment, and Onodera’s prognostic nutritional index (PNI) [10] and Physiological and Operative Severity Score for the enUmeration of Mortality and morbidity (POSSUM) [11] are also reported to be useful risk predictors for gastrectomy [12, 13]. It is also recommended that extent of gastrectomy and lymphadenectomy be limited to avoid complications for elderly patients [14].

This study aimed to retrospectively investigate the prognostic factors for elderly gastric cancer patients aged ≥ 80 years who underwent gastrectomy. The results may be helpful in predicting what characteristics and procedures are associated with better prognosis and to decide surgical indications for these patients.

## Methods

### Patients and data retrieval

**Fig. 1:**
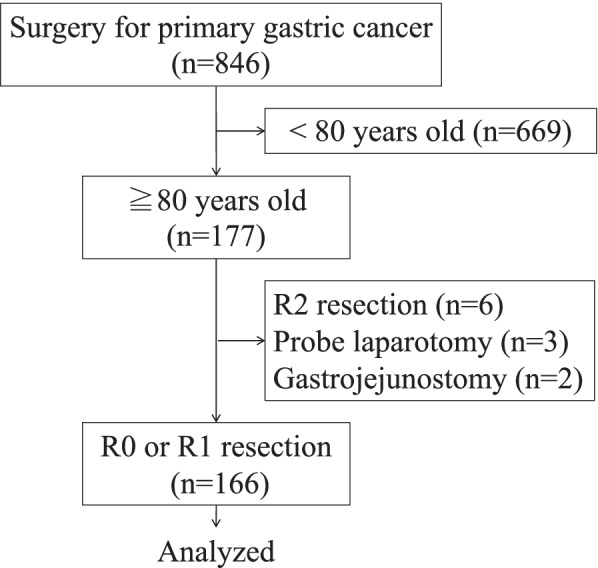
The participant flow diagram. R2, macroscopic residual tumor; R1, microscopic residual tumor; R0, no residual tumor

The medical records of 112 and 65 consecutive patients aged ≥ 80 years who underwent surgery for gastric cancer at Kawasaki Medical School Hospital between 2010 and 2019 and at Kawasaki Medical School General Medical Center between 2011 and 2019, respectively, were retrospectively reviewed. Excluding six patients who underwent R2 (macroscopic residual tumor) resection, three who underwent probe laparotomy, and two who underwent gastrojejunostomy, 166 patients who underwent R0 (curative) or R1 (microscopic residual tumor) resection were analyzed. A flowchart of the patient selection is shown in Fig. 1.

The following information was collected from the patients’ medical records: age, sex, ECOG-PS score, ASA-PS classification, PNI, physiological score of POSSUM, surgical procedure, histological classification of gastric cancer, clinicopathological cancer stage, residual tumor, postoperative complications, postoperative chemotherapy, and prognosis. The effects of these preoperative characteristics, perioperative treatment, and pathological features on overall survival (OS) were evaluated by univariate and multivariate analyses. The PNI is calculated using the following formula: 10 × serum albumin (g/dL) + 0.005 × total lymphocyte count (/mm^3^). The POSSUM physiological score was calculated based on the patient’s age, cardiac signs, chest radiography signs, respiratory history, systolic blood pressure, pulse rate, Glasgow coma scale score, hemoglobin level, white blood cell count, plasma urea level, plasma sodium level, plasma potassium level, and electrocardiography results. Each item was scored from 1 (normal) to 8 (abnormal). Adding the scores gives a physiological score ranging from a minimum of 12 to a maximum of 88, with a higher score indicating a higher surgical risk. The clinicopathological findings of gastric cancer are recorded according to the Japanese classification of gastric carcinoma: 3rd English edition [15], and the surgical procedure and lymphadenectomy are recorded according to the Japanese Gastric Cancer Treatment Guidelines 2018 (5th edition) [16]. Prognoses including the last date known to be alive or the date and cause of death were gathered from the medical records at the participating institutions or referral institutions, the condolences sections of local newspapers, or by calling the patients or their families.

### Statistical analysis

OS was defined as the interval from the date of surgery to the date of death from any cause. Surviving patients were censored at the date they were last known to be alive. Hazard ratios (HRs) for death were estimated using the Cox regression analysis. Analyses were performed using the JMP software (version 14.2.0 for Windows; SAS Institute Inc., Cary, NC, USA).

## Results

The patients’ characteristics are summarized in Table 1. The oldest patient in the current series was a 96-year-old male. Seventy-seven percent of participating patients were ECOG-PS 0,1, and 72% of them were ASA-PS 1,2. The extents of gastrectomy and lymphadenectomy were limited in some cases. Proximal gastrectomy was performed in 11 patients, although seven of them were cT2-4 and/or cN+. Local gastrectomy was selected in 13 patients, including 4, 4, and 5 patients with cT1aN0, cT1bN0, and cT2N0, respectively. Limited lymphadenectomy less than D2 was applied for 127 patients, although 70 of them were cT2-4 and/or cN+. One hundred and fifty-one patients underwent R0 resection, while 15 patients underwent R1 resection including 11 patients with CY1 and five patients with positive resection margins (one patient with both CY1 and positive resection margin). After surgery, 10 patients received chemotherapy: S-1 monotherapy for nine patients (1, 1, 3, 2, and 2 patients with pStage IIA, IIB, IIIA, IIIb, and IV, respectively) and S-1 plus cisplatin for one patient (pStage IV).

**Table 1:** Patients’ characteristics

Age, years	
Median (range)	83 (80–96)
Sex, *n* (%)	
Male	113 (68)
ECOG-PS score, *n* (%)	
0	81 (49)
1	47 (28)
2	28 (17)
3	10 (6)
ASA-PS, *n* (%)	
1	7 (4)
2	112 (67)
3	45 (27)
4	2 (1)
PNI	
Median (range)	44.5 (19.1–63.0)
POSSUM physiological score	
Median (range)	28 (20–46)
Approach, *n* (%)	
Open	120 (72)
Laparoscopic	46 (28)
Extent of gastrectomy, *n* (%)	
Distal	104 (63)
Total	32 (19)
Proximal	11 (7)
Local	13 (8)
Completion	5 (3)
Subtotal of remnant	1 (1)
Extent of lymphadenectomy, *n* (%)	
D2+	1 (1)
D2	38 (23)
D1+	73 (44)
D1	37 (22)
D0	17 (10)
Histological classification, *n* (%)	
tub	93 (56)
pap	10 (6)
por	45 (27)
sig	5 (3)
muc	7 (4)
other	6 (4)
pStage, *n* (%)	
IA	59 (36)
IB	12 (7)
IIA	30 (18)
IIB	19 (11)
IIIA	18 (11)
IIIB	13 (8)
IIIC	2 (1)
IV	13 (8)
Residual tumor, *n* (%)	
R0	151 (91)
R1	15 (9)
Postoperative chemotherapy, *n* (%)	
No	156 (94)
Yes	10 (6)

Pathological findings are provided according the Japanese classification of gastric carcinoma: 3rd English edition. *ECOG-PS* Eastern Cooperative Oncology Group Performance Status, *ASA-PS* American Society of Anesthesiologists physical status, *PNI* Onodera's prognostic nutritional index, *POSSUM* Physiological and Operative Severity Score for the enUmeration of Mortality and morbidity

Postoperative complications of grade II or worse according to the Clavien–Dindo classification [17] were detected during hospitalization in 45 patients (27%). Details of the surgical and medical complications are shown in Table 2. Anastomotic leakage (*n* = 6, 3.6%) and abscess (*n* = 6) were the most common surgical complications and resulted in death for one patient each. Pneumonia (*n* = 10, 6.0%) and other respiratory failure (*n* = 5, 3.0%) were the most and second most common medical complications, respectively. Eight such patients needed mechanical ventilation, and two died during hospitalization.

**Table 2:** Postoperative complications ≥ Clavien-Dindo II

	Total	Clavien-Dindo					
		II	IIIA	IIIB	IVA	IVB	V
Surgical complications	20						
Anastomotic leakage	6	3	1		1		1
Anastomotic bleeding	1	1					
Anastomotic stenosis	1	1					
Pancreatic fistula	4	1	2	1			
Abscess	6	2	2			1	1
Bowel obstruction	1			1			
Wound dehiscence	1			1			
Medical complications	30						
Stroke	4	3					1
Myoclonus	1	1					
Supraventricular arrhythmia	3	3					
Ischemic heart disease	1	1					
Pneumonia	10	6			2		2
Other respiratory failure	5		1		4		
Pleural effusion	2		2				
Pseudomembranous colitis	2	2					
Liver damage	2	2					
Influenza infection	1	1					
Urinary tract infection	2	2					
Sepsis	1						1
No	121						

The total number of complications and patients do not match because some patients had multiple complications

At the time of analysis, 72 patients had died. The causes of death are shown in Table 3. The leading cause of death was gastric cancer (37% of known cause), followed by pneumonia (21%), stroke (11%) and senility (11%). The median OS time was 62.3 months and the 5-year OS rate was 51.4%.

**Table 3:** Cause of death

Variable	(*n* = 72)
Gastric cancer	24
Surgical complication	2
Stroke	7
Cardiovascular disease	4
Pneumonia	14
Other respiratory disease	2
Bowel obstruction	1
Other malignancy	3
Trauma	1
Senility	7
Unknown cause	7

In univariate analyses, age (≥ 90 vs. ≥ 80, < 85), ECOG-PS (3 vs. 0), ASA-PS (3, 4 vs. 1, 2), PNI (< 40 vs. ≥ 45), POSSUM physiological score (≥ 40 vs. ≥ 20, ≤ 29), surgical approach (laparoscopic vs. open), extent of gastrectomy [total (including completion gastrectomy), proximal vs. distal (including subtotal resection of remnant stomach)], extent of lymphadenectomy (D1 vs. ≥ D2), pathological stage (II–IV vs. I), and residual tumor (R1 vs. R0) were significantly correlated with worse OS (Table 4). Multivariate analysis conducted with these significant factors of age, ECOG-PS, ASA-PS, PNI, POSSUM physiological score, surgical approach, extent of gastrectomy, extent of lymphadenectomy, pathological stage, and residual tumor revealed that ASA-PS (3,4 vs. 1,2), extent of gastrectomy (total, proximal vs. distal), extent of lymphadenectomy (D0 vs. ≥ D2), and pathological stage (II–IV vs. I) were independent risk factors for mortality.

**Table 4:** Univariate and multivariate analyses of hazard ratios for death

Valuables	*n*	MST (months)	Univariate analysis			Multivariate analysis		
			HR	(95%CI)	*p*	HR	(95%CI)	*p*
Age (year)								
≥ 80, < 84	101	> 120	Reference			Reference		
≥ 85, < 89	53	77.5	1.16	(0.68–1.96)	0.59	0.89	(0.49–1.65)	0.72
≥ 90	12	29.7	2.55	(1.30–5.00)	< 0.01	2.09	(0.87–5.03)	0.10
Sex								
Male	113	62.3	Reference					
Female	53	> 109	1.04	(0.63–1.71)	0.89			
ECOG-PS								
0	81	85.9	Reference			Reference		
1	47	48.5	1.25	(0.71–2.21)	0.44	0.85	(0.44–1.65)	0.64
2	28	28.7	1.74	(0.94–3.25)	0.08	1.54	(0.75–3.15)	0.24
3	10	14.9	3.75	(1.62–8.67)	< 0.01	1.44	(0.47–4.38)	0.52
ASA-PS								
1,2	118	85.9	Reference			Reference		
3,4	47	20.8	2.60	(1.58–4.28)	< 0.01	2.30	(1.24–4.27)	< 0.01
PNI								
≥ 45	77	> 120	Reference			Reference		
≥ 40, < 45	35	77.5	1.52	(0.80–2.91)	0.20	1.82	(0.87–3.79)	0.11
< 40	53	22.7	2.62	(1.54–4.45)	< 0.01	1.39	(0.68–2.86)	0.36
POSSUM physiological score								
≥ 20, ≤ 29	99	79.2	Reference			Reference		
≥ 30, ≤ 39	56	54.0	1.13	(0.68–1.88)	0.63	0.68	(0.38–1.20)	0.18
≥ 40	11	6.7	3.95	(1.89–8.24)	< 0.01	2.00	(0.78–5.16)	0.15
Cardiac signs and chest radiography signs								
No failure	34	70.2	Reference					
Diuretic, digoxin, antianginal or hypertensive therapy	61	55.6	1.06	(0.57–1.98)	0.85			
Peripheral edema, warfarin therapy, borderline cardiomegaly	60	74.1	1.03	(0.55–1.94)	0.93			
Raised jugular venous pressure, cardiomegaly	11	36.6	1.64	(0.63–4.27)	0.31			
Respiratory history								
No dyspnea	69	51.2	Reference					
Dyspnea on exertion, mild COPD	75	86.7	0.69	(0.42–1.13)	0.14			
Limiting dyspnea, moderate COPD	9	29.7	2.18	(1.00–4.76)	0.05			
Dyspnea at rest, fibrosis or consolidation	13	24.3	2.13	(0.93–4.88)	0.07			
Surgical approach								
Open	120	40.2	Reference			Reference		
Laparoscopic	46	85.9	0.45	(0.25–0.82)	< 0.01	1.58	(0.64–3.89)	0.32
Extent of gastrectomy								
Distal^a^	105	85.9	Reference			Reference		
Total^b^	37	20.8	2.40	(1.42–4.05)	< 0.01	2.17	(1.10–4.31)	0.03
Proximal	11	29.3	2.31	(1.07–4.99)	0.03	4.05	(1.45–11.3)	< 0.01
Local	13	> 97.6	0.79	(0.31–2.02)	0.62	0.08	(0.01–0.90)	0.04
Extent of lymphadenectomy								
≥ D2	39	> 120	Reference			Reference		
D1+	73	77.5	1.20	(0.62–2.33)	0.58	1.42	(0.66–3.04)	0.37
D1	37	22.4	2.56	(1.30–5.04)	< 0.01	2.00	(0.90–4.41)	0.09
D0	17	62.3	1.13	(0.45–2.84)	0.80	12.4	(1.58–97.7)	0.02
Histological classification								
tub,pap	103	77.5	Reference					
pos,sig	50	28.6	1.63	(0.99–2.68)	0.05			
other	13	> 109	0.95	(0.38–2.41)	0.91			
pStage								
I	71	85.9	Reference			Reference		
II	49	48.5	2.00	(1.09–3.67)	0.03	2.39	(1.03–5.56)	0.04
III	33	20.8	3.16	(1.68–5.95)	< 0.01	3.03	(1.24–7.40)	0.02
IV	13	6.1	9.93	(4.45–22.2)	< 0.01	5.44	(1.17–25.4)	0.03
Residual tumor								
R0	151	68.5	Reference			Reference		
R1	15	11.5	2.92	(1.48–5.73)	< 0.01	2.11	(0.48–9.40)	0.33
Postoperative complication ≥ CD II								
No	121	68.5	Reference					
Yes	45	44.8	1.46	(0.88–2.44)	0.14			
Postoperative chemotherapy								
No	156	62.3	Reference					
Yes	10	> 83.4	1.37	(0.55–3.41)	0.50			

Cardiac signs and chest radiography signs and respiratory history are classified according to the POSSUM scoring system. Pathological findings are provided according the Japanese classification of gastric carcinoma: 3rd English edition

^a^Distal gastrectomy includes one case with subtotal resection of remnant stomach

^b^Total gastrectomy includes five cases with completion gastrectomy of remnant stomach

*MST* median survival time, *HR* hazard ratio, *CI* confidence interval, *ECOG-PS* Eastern Cooperative Oncology Group Performance Status, *ASA-PS* American Society of Anesthesiologists physical status, *PNI* Onodera’s prognostic nutritional index, *POSSUM* Physiological and Operative Severity Score for the enUmeration of Mortality and morbidity, *COPD* chronic obstructive pulmonary disease, *CD* Clavien-Dindo

## Discussion

In the current retrospective study, we analyzed the surgical outcomes of 166 consecutive patients aged ≥ 80 years with gastric cancer who underwent gastrectomy with curative intent in two institutions. Most of the participants were in relatively good general condition, which may mean that only selected patients underwent surgery. The extents of gastrectomy and lymphadenectomy were often limited. The incidence of postoperative respiratory complications including pneumonia was high, and complications were likely to become serious. Pneumonia was the second leading cause of death following gastric cancer. Univariate analyses showed that extremely advanced age (≥ 90 years), worse general, physical, nutritional, or physiological condition (ECOG-PS 3, ASA-PS 3,4, PNI < 40, POSSUM physiological score ≥ 40), open surgery, total or proximal gastrectomy, D1 lymphadenectomy, advanced cancer stage (pStage II–IV), and R1 resection were prognostic factors for worse OS. A multivariate analysis revealed that ASA-PS 3,4, total or proximal gastrectomy, D0 lymphadenectomy, and pStage II–IV were independent risk factors for worse OS.

This result may mean that, among several parameters that may predict postoperative mortality, ASA-PS, while simple, is the keenest classification. A disadvantage of ASA-PS is that it can vary among evaluators even for the same patient. To address this, specific examples and explanations were added in 2014 as follows [9]. The definition of ASA-PS 3 is “a patient with severe systemic disease”; for example, poorly controlled diabetes mellitus or hypertension, chronic obstructive pulmonary disease, morbid obesity (body mass index ≥ 40), active hepatitis, alcohol dependence or abuse, implanted pacemaker, moderate reduction of ejection fraction, end-stage renal disease undergoing regularly scheduled dialysis, history (> 3 months) of myocardial infarction, cerebrovascular accident, transient ischemic attack, or coronary artery disease stents. Since this edition, ASA-PS has been evaluated relatively objectively. Which factor of ASA-PS influenced the prognosis was unclear in this study. Instead, we collected data on cardiac signs, chest radiography signs, and respiratory history to calculate the POSSUM physiological scores. Although these factors themselves were not prognostic factors, moderate or severe respiratory disease was associated with poor prognosis.

The extent of gastrectomy was an independent risk factor for mortality. Patients who underwent gastrectomy including the cardia (total, proximal, and completion gastrectomy) had shorter survival than those who underwent distal gastrectomy or subtotal resection of the remnant stomach. The former patients were more likely to die of pneumonia and senility (six and four among 48 patients, respectively) than were the latter patients (six and three among 105 patients, respectively), although no significant differences were found. Preserving the cardia may contribute to preventing regurgitation and malnutrition after gastrectomy. A previous report with patients aged ≥ 85 years also mentioned that the prognosis of patients undergoing total gastrectomy was worse than that of patients after distal gastrectomy [18].

As well as the extent of gastrectomy, the extent of lymphadenectomy is also recommended to be limited in patients aged ≥ 80 years [19]. A recent paper showed that D2 lymphadenectomy was an independent risk factor for postoperative pneumonia in patients aged ≥ 75 years [20]. In our study, ≥ D2 lymphadenectomy provided fair prognoses, and D1+ lymphadenectomy was also acceptable. However, D0 lymphadenectomy was an independent risk factor for worse OS compared with D2 lymphadenectomy, which may mean that excessively limited lymphadenectomy for advanced cancer is best avoided.

The pros and cons of adjuvant chemotherapy for gastric cancer in the elderly are also controversial. In the ACTS-GC trial [21], which showed the effectiveness of S-1 adjuvant treatment for stage II or III gastric cancer, the eligibility criteria excluded patients aged over 80 years. In the current series, the number of patients who received postoperative chemotherapy was only 10 (6%), which was too small for a statistical analysis. A questionnaire survey of JCOG also showed that only 99 (6.0%) of 1660 gastrectomized patients aged > 80 years received S-1 adjuvant chemotherapy [22]. A phase III trial to confirm modified S-1 adjuvant chemotherapy for pathological stage II/III vulnerable elderly gastric cancer patients after gastric resection (JCOG1507, BIRDIE) is ongoing [23], and the results are awaited.

The present study had several potential limitations. First, this study was limited by its retrospective nature. Second, some patients were not followed up for a sufficient period of time. Third, this study was conducted with a relatively small number of patients from two institutions. We would like to collect and reexamine more patients from more institutions in the future.

## Conclusion

We retrospectively analyzed the prognostic factors for gastric cancer patients aged ≥ 80 years after gastrectomy. The multivariate analyses showed that ASA-PS 3,4, gastrectomy including cardia, D0 lymphadenectomy, and pathological stage II-IV had worse prognoses. Gastrectomy for elderly patients with severe systemic disease should be carefully performed, and the gastric cardia should be preserved if possible.

## Data Availability

The datasets used and analyzed during this study are available from the corresponding author upon reasonable request.

## Abbreviations

ASA-PS: American Society of Anesthesiologists physical status
HR: Hazard ratio
CI: Confidence interval
NCD: National Clinical Database
ECOG-PS: Eastern Cooperative Oncology Group performance status
POSSUM: Physiological and Operative Severity Score for the enUmeration of Mortality and morbidity
PNI: Prognostic nutritional index
OS: Overall survival
